# Inzidenz, Behandlung und Prognose des Prostatakarzinoms in Baden-Württemberg: Analyse anhand von Krebsregisterdaten

**DOI:** 10.1007/s00120-024-02275-w

**Published:** 2024-02-05

**Authors:** Thomas Stefan Worst, Irina Surovtsova, Tilo Vogel, Martin Zauser, Manuel Christian Neuberger, Frederik Wessels, Maurice Stephan Michel, Philipp Nuhn, Philipp Morakis

**Affiliations:** 1 Klinik für Urologie und Urochirurgie, Universitätsmedizin Mannheim, Theodor-Kutzer-Ufer 1–3, 68167 Mannheim, Deutschland; 2 Klinische Landesregisterstelle GmbH, Krebsregister Baden-Württemberg, Birkenwaldstraße 149, 70191 Stuttgart, Deutschland; 3 Geschäftsstelle Qualitätskonferenzen bei der Klinischen Landesregisterstelle GmbH, Krebsregister Baden-Württemberg, Birkenwaldstraße 149, 70191 Stuttgart, Deutschland; 4 Klinik für Urologie Kiel, Universitätsklinikum Schleswig-Holstein, Arnold-Heller-Straße 3, 24105 Kiel, Deutschland

**Keywords:** Real-World-Daten, Krebsregister, Therapieverfahren, Radikale Prostatektomie, Roboterassistierte Chirurgie, Real world data, Tumor register, Therapeutic procedures, Radical prostatectomy, Robot-assisted surgery

## Abstract

**Hintergrund:**

Das Prostatakarzinom (PCa) ist die häufigste solide Tumorerkrankung des Mannes in Deutschland. Die Erfassung epidemiologischer und klinischer Daten erfolgt seit mehreren Jahren aufgrund gesetzlicher Vorgaben zentralisiert über die Landeskrebsregister. Damit ist die Meldung von Erstdiagnosen (ED), Therapien und Verläufen von Krebserkrankungen in Deutschland verpflichtend. Entsprechend der Fragestellungen der Behandler müssen diese Daten aufbereitet werden.

**Ziele:**

Intention dieser Arbeit war die Darstellung der Entwicklung von Neuerkrankungen, Erkrankungsstadien, Behandlungsverfahren und Prognose des PCa in Baden-Württemberg (BW).

**Methoden:**

Ausgewertet wurden hierfür die Daten des Krebsregisters BW von Patienten mit PCa in den ED-Jahren 2013 bis 2021. Die Auswertung erfolgte mittels deskriptiver Statistik, χ^2^-Test und Kaplan-Meier-Analysen.

**Ergebnisse:**

Gemeldet wurden 84.347 PCa-ED. Bei 55,3 % der Patienten lag das klinische Stadium und bei 75,7 % das ISUP-Grading vor. Bis 2019 zeigte sich ein Anstieg der ED. Der Anteil primär metastasierter Erkrankungen war rückläufig (2013: 19,6 %, 2021: 12,0 %), der Anteil lokalisierter Tumoren (2013: 65,5 %, 2021: 77,1 %) nahm zu. Bei der Therapie lokal begrenzter Tumoren dominierte die radikale Prostatektomie (RP) mit im Mittel 60,1 %. Der Anteil der roboterassistierten Operationen stieg von 23,7 % (2013) auf 60,8 % (2021) bei einem Rückgang der R1-Rate von 34,8 % auf 26,2 %. Das progressionsfreie Überleben korrelierte mit dem Tumorstadium und der ISUP-Gruppe.

**Schlussfolgerung:**

Es zeigte sich ein Anstieg der gemeldeten PCa-Fälle, wobei der Anteil der spät diagnostizierten Tumoren abnahm. Die Behandlung erfolgte in lokalisierten Stadien zumeist operativ, mit steigendem Anteil der roboterassistierten RP. Langfristig prognoseentscheidend sind die frühzeitige Diagnosestellung und Behandlung.

**Graphic abstract:**

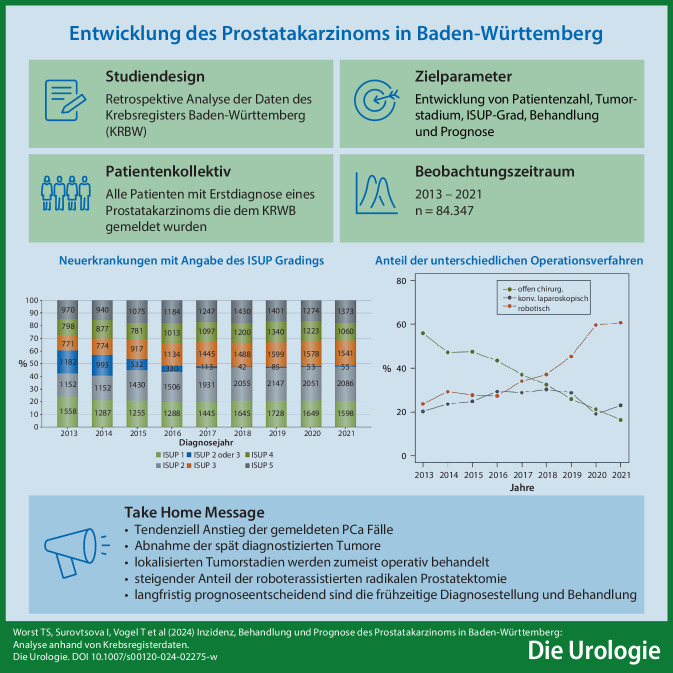

## Abstract (en)

**Background:**

Prostate cancer (PCa) is the most common solid tumor in men in Germany. Collection of epidemiological and clinical data has been centralized for several years due to legal requirements via the state cancer registries. Thus, the reporting of diagnosis, therapy, and progression of cancer is obligatory in Germany. These data needs to be processed based on the questions of the treating physicians.

**Objectives:**

Intention of this work was to present the development of new cases, disease stages, treatment procedures and prognosis of PCa in Baden–Württemberg (BW).

**Methods:**

For this purpose, data of the cancer registry BW regarding patients with PCa first diagnosed between 2013 and 2021 were evaluated. The evaluation was performed using descriptive statistics, Χ^2^ test and Kaplan–Meier analysis.

**Results:**

A total of 84,347 new diagnoses of PCa were reported. Clinical stage was present in 55.3% of patients. Assignment by International Society of Urological Pathology (ISUP) groups was present in 75.7%. A steady increase in primary diagnosis was evident through 2019. The proportion of primary metastatic disease decreased (2013: 19.6% vs. 2021: 12.0%), and the proportion of localized tumors increased (2013: 65.5% vs. 2021: 77.1%). Radical prostatectomy (RP) dominated the treatment of localized tumors with a mean of 60.1%. The proportion of robot-assisted surgery increased from 23.7% (2013) to 60.8% (2021) with a decrease in the R1 rate from 34.8 to 26.2%. Progression-free survival correlated closely with tumor stage and ISUP group.

**Conclusion:**

An increase in PCa cases and a decrease of advanced tumors were observed. Treatment was mostly surgical in localized stages, with increasing proportion of robotic-assisted RP. Early diagnosis and treatment are critical for long-term prognosis.

## Einleitung

Das Prostatakarzinom (PCa) ist die zweithäufigste maligne Tumorerkrankung bei Männern weltweit und die häufigste in Deutschland [1, 2]. Es bestehen regionale Unterschiede sowohl hinsichtlich der Inzidenz als auch der Mortalität des PCa. Die Inzidenz ist besonders hoch in Nordeuropa, Amerika und Australien [2–4]. Im europäischen Vergleich befindet sich Deutschland im Mittelfeld mit einer Inzidenz von jährlich 99,1 neuen Fällen pro 100.000 Einwohner. Dies resultierte im Jahr 2018 in 65.200 diagnostizierten Neuerkrankungen [2]. Im gleichen Zeitraum starben 14.963 Männer an einem PCa. Auch in Deutschland gibt es – wenn auch geringe – regionale Variationen hinsichtlich der Inzidenz des PCa: im Zeitraum 2017–2018 war sie am höchsten in Sachsen-Anhalt und am niedrigsten in Thüringen [2].

Aufgrund der demografischen Entwicklung mit einem immer größer werdenden Anteil der älteren Bevölkerung ist auch in Deutschland eine Zunahme der PCa-Diagnosen zu erwarten. Schätzungen in den USA gehen von einer Zunahme der Neudiagnosen um > 25 % bis zum Jahre 2040 aus [5]. Dies unterstreicht sowohl die medizinische als auch die gesundheitspolitische Relevanz dieser Erkrankung.

Mit Inkrafttreten des Bundeskrebsregistergesetzes im Jahre 1995 wurden die Bundesländer verpflichtet, bevölkerungsbezogene Krebsregister einzurichten. Seit dem Jahr 2000 obliegt die Krebsregistergesetzgebung dem jeweiligen Landesrecht. 2006 wurde ein neues Landeskrebsregistergesetz für Baden-Württemberg (LKrebsRG) verabschiedet. Erstmalig in Deutschland bestand damit die Verpflichtung zur Sammlung klinischer Daten und deren Auswertung im Hinblick auf die klinische Versorgungslage im Land. 2009 erfolgte die Aufnahme der Registrierung in BW. Diese wurde stufenweise eingeführt: 01/2009: Meldepflicht Stufe I für onkologische Schwerpunkte und Tumorzentren, 07/2011: Meldepflicht Stufe II für Krankenhäuser und pathologische Einrichtungen sowie 10/2011: Meldepflicht Stufe III für niedergelassene Ärzte.

Gegenwärtig existieren in Deutschland für maligne Erkrankungen bei Erwachsenen 15 Krebsregister, je eines in jedem Bundesland. Lediglich die Bundesländer Berlin und Brandenburg haben ein gemeinsames Krebsregister. Weiterhin existiert das bundesweit zuständige Kinderkrebsregister in Mainz [6]. Auf nationaler Ebene werden die in den Krebsregistern erhobenen Daten im Zentrum für Krebsregisterdaten des Robert-Koch-Instituts in Berlin zusammengeführt [7]. Während im Gebiet der neuen Bundesländer bereits vor der Wiedervereinigung systematisch Daten von onkologischen Patienten in einem Krebsregister gesammelt wurden, liegen vergleichbare Daten in den alten Bundesländern zumeist erst seit ca. 20 bis 25 Jahren vor. Hierbei hat sich die Methodik der Erfassung und deren Detailtiefe in den vergangenen 20 Jahren sukzessive verbessert.

Ziel dieser Untersuchung war es, die seit 2013 beim Krebsregister Baden-Württemberg (KRBW) eingegangenen Daten bei Patienten mit PCa entsprechend der Anforderungen aufzuarbeiten. Diese Auswertung soll damit einen Beitrag zur Verbesserung der Versorgung von Patienten mit PCa in BW und in ganz Deutschland leisten.

## Methodik

Es erfolgte die Auswertung aller für die Jahre 2013 bis 2021 an das KRBW gemeldeten PCa-Erstdiagnosen (ED; Datenbankstand vom 01.07.2023). Vor Auswertung der Daten wurden entsprechende Plausibilitäts- und Konsistenzchecks vorgenommen.

Es erfolgte die Gruppierung und Substratifizierung der Daten entsprechend ihren klinischen Parametern. Zur Anwendung kamen deskriptive statistische Analysen, weiterhin der χ^2^-Test sowie Kaplan-Meier-Analysen. Die Auswertung erfolgte mittels „tidyverse“, „survival“ und „survminer“ Pakete in R Software Version 4.1.1 (https://www.r-project.org/). *p*-Werte < 0,05 wurden als signifikant gewertet.

## Ergebnisse

### Epidemiologie und Datenkonsistenz von 2013 bis 2021

Insgesamt wurden in den Jahren 2013 bis 2021 84.347 PCa-ED an das KRBW gemeldet. Tab. 1 gibt eine Aufschlüsselung der ED pro Jahr. In den Jahren 2015 bis 2019 war ein sukzessiver Anstieg der ED zu verzeichnen mit einem Maximum von 10.789 gemeldeten ED in 2019. In den Jahren 2020 und 2021 lag die Zahl der gemeldeten ED etwas geringer. Angegeben sind Alter, ISUP (International Society of Urological Pathology) Grading, klinisches Stadium und die Risikogruppen nach D’Amico.

**Tab. 1:** Prostatakarzinom (PCa)-Erstdiagnosen (ED) nach Diagnosejahren sowie der Anteil der Patienten mit Angabe von klinischen Tumorstadien, ISUP-Gruppen und D’Amico-Risikogruppen

Diagnosejahr	2013	2014	2015	2016	2017	2018	2019	2020	2021	Gesamt
*Gesamt* *(%)*	8.596 (100)	8.174 (100)	8.075 (100)	9.062 (100)	9.623 (100)	9.931 (100)	10.789 (100)	9.794 (100)	10.303 (100)	*84.347 (100)*
*Mittleres Alter (SD)*	69,6 (8,6)	69,9 (8,9)	70,0 (9,0)	70,3 (8,8)	70,5 (8,9)	70,6 (9,1)	70,7 (8,9)	70,6 (8,9)	70,7 (8,9)	*70,4* *(8,9)*
*Mit ISUP* *(%)*	6.431 (74,8)	6.023 (73,7)	5.990 (74,2)	6.455 (71,2)	7.278 (75,6)	7.860 (79,1)	8.300 (76,9)	7.828 (79,9)	7.713 (74,9)	*63.878 (75,7)*
*Mit klinischem Stadium* *(%)*	3.628 (42,2)	3.765 (46,1)	3.964 (49,1)	4.528 (50,0)	5.525 (57,4)	6.272 (63,2)	6.630 (61,5)	6.140 (62,7)	6.223 (60,4)	*46.675* *(55,3)*
*Lokal begrenzt* *(% von „mit klinischem Stadium“)*	2.375 (65,5)	2.403 (63,8)	2.591 (65,4)	3.160 (69,8)	4.043 (73,2)	4.587 (73,1)	4.901 (73,9)	4.653 (75,8)	4.797 (77,1)	*33.510* *(71,8)*
*Mit D’Amico* *(% von „lokal begrenzt“)*	1.973 (83,1)	1.940 (80,7)	2.034 (78,5)	2.443 (77,3)	3.208 (79,3)	3.736 (81,4)	4.004 (81,7)	3.905 (83,9)	3.771 (78,6)	*27.014 (80,6)*

*ISUP* Internationale Gesellschaft für Urologische Pathologie

**Abb. 1:**
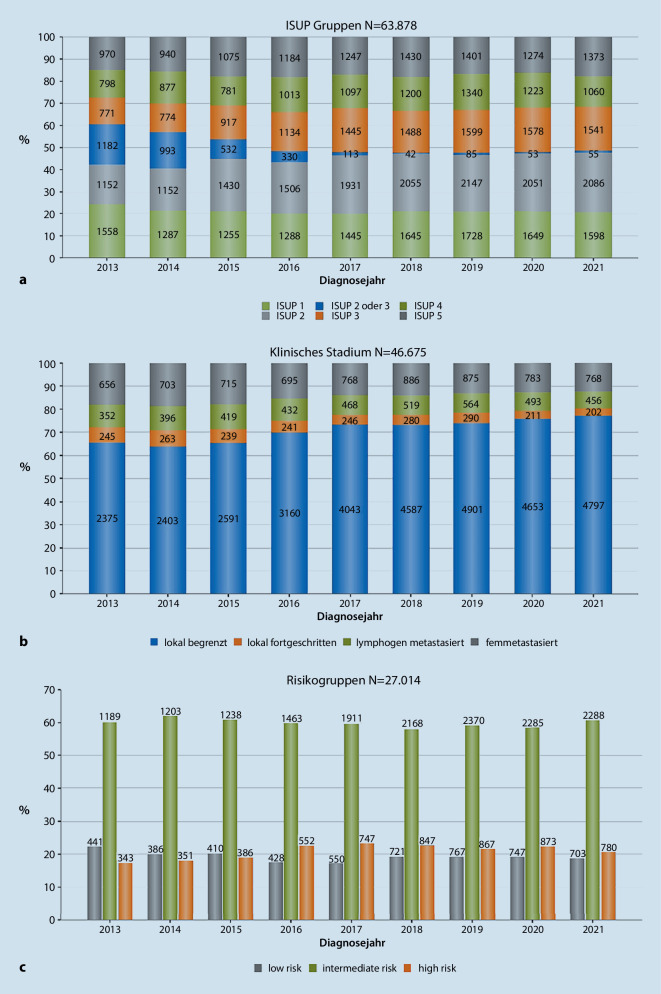
**a** Verteilung der verschiedenen ISUP-Gruppen (Internationale Gesellschaft für Urologische Pathologie) und **b** der klinischen Tumorstadien bezogen auf alle Tumoren sowie **c** der D’Amico-Risikogruppen bezogen auf lokal begrenzte Tumoren über die Diagnosejahre im Erfassungszeitraum

Nicht für alle Patienten wurden vollständige Datensätze an das KRBW übermittelt. Bei 63.878 Patienten lagen Informationen zum ISUP-Grading vor (75,7 %). Während der Anteil der ISUP-1-Patienten von 2013 bis 2021 von 24,2 % auf 20,7 % leicht rückläufig war, nahm der Anteil an Patienten mit ISUP-2-Tumoren von 17,9 % im Jahr 2013 auf 27,1 % im Jahr 2021 deutlich zu (Abb. 1a). Dies geht einher mit einem Rückgang des Anteils der Patienten, bei denen aufgrund einer fehlenden Angabe des prädominanten Gleason-Patterns keine Zuordnung zu ISUP 2 oder 3 möglich war. Im Jahr 2013 lag ihr Anteil noch bei 18,4 %. Dieser konnte bis zum Jahr 2021 auf 0,7 % gesenkt werden. Dies ist ein Indikator für eine zunehmende Datenkonsistenz.

Das klinische Stadium, im Sinne einer Angabe die eine Einteilung in die Gruppen „lokal begrenzt“, „lokal fortgeschritten“, „lymphogen metastasiert“ und „fernmetastasiert“ ermöglichte, lag bei 46.675 Patienten (55,3 % aller Patienten) vor. Erfreulicherweise stieg der Anteil der Patienten mit entsprechender Dokumentation jedoch deutlich von 42,2 % in 2013 auf 60,4 % in 2021 an. Beim überwiegenden Anteil handelte es sich – bezogen auf die Patienten mit vorliegender Dokumentation – um lokal begrenzte Tumoren (Abb. 1b). Lokal fortgeschrittene, lymphogen metastasierte und fernmetastasierte Tumoren machten einen entsprechend geringeren Anteil aus.

Während der relative Anteil der lokal begrenzten Tumoren in den Jahren 2013 bis 2021 von 65,5 % auf 77,1 % zunahm, nahm der Anteil der weiter fortgeschrittenen Tumoren entsprechend ab. Insbesondere der Anteil der fernmetastasierten Tumoren sank von 18,1 % in 2013 auf 12,3 % in 2021.

Die D’Amico-Risikogruppe konnte bei 27.014 Patienten eindeutig bestimmt werden (32,0 % der Gesamtkohorte bzw. 80,6 % der als lokal begrenzt dokumentierten Tumoren). Der Anteil der Tumoren mit intermediärem Risiko zeigte eine recht konstante Verteilung über den Erfassungszeitraum (Abb. 1c). Der Anteil der Niedrigrisikotumore sank von 22,4 auf 18,6 %, der Anteil der Hochrisikotumore stieg von 17,4 auf 20,7 %.

Werden die Parameter klinisches Tumorstadium und ISUP-Gruppe aller Patienten, bei denen diese beiden Parameter vorliegen (*n* = 41.034), zueinander in Bezug gesetzt, zeigt sich eine deutliches umgekehrt proportionales Verhältnis (χ^2^: *p* < 0,001; Tab. 2).

**Tab. 2:** Kreuztabelle von Tumorstadien und ISUP-Grading

	ISUP 1	ISUP 2	ISUP 2/3	ISUP 3	ISUP 4	ISUP 5
*Lokal begrenzt (%)*	7.656 (25,1 %)	9.160 (30,0 %)	1.722 (5,6 %)	5.874 (19,2 %)	3.643 (11,9 %)	2.471 (8,1 %)
*Lokal fortgeschritten (%)*	71 (3,6 %)	231 (11,6 %)	115 (5,8 %)	392 (19,6 %)	544 (27,3 %)	642 (32,2 %)
*Lymphogen metastasiert (%)*	74 (2,1 %)	287 (8,2 %)	140 (4,0 %)	600 (17,2 %)	864 (24,7 %)	1.530 (43,8 %)
*Fernmetastasiert (%)*	97 (1,9 %)	178 (3,5 %)	110 (2,2 %)	412 (8,2 %)	1.263 (25,2 %)	2.958 (58,9 %)

*ISUP* Internationale Gesellschaft für Urologische Pathologie

## Anteil der unterschiedlichen Behandlungsalternativen

**Abb. 2:**
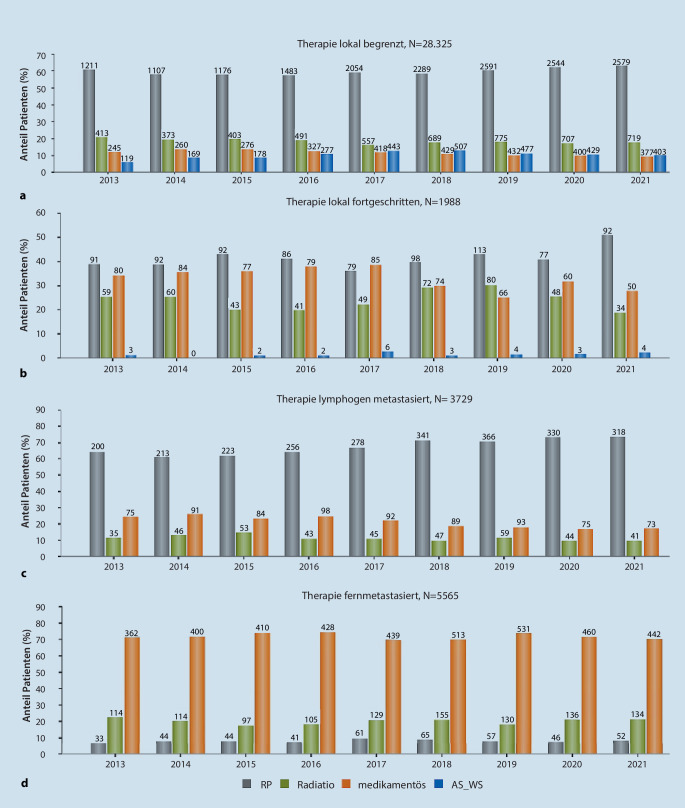
Verteilung der unterschiedlichen Behandlungsstrategien radikale Prostatektomie (RP), Radiatio, medikamentöse Therapie und abwartendes Vorgehen mittels Active Surveillance (AS) oder Watchful Waiting bzw. Wait and See (WS) bei Patienten mit **a** lokal begrenztem, **b** lokal fortgeschrittenem, **c** lymphogen metastasiertem (+ *n* = 21 mit WS nicht abgebildet) und **d** fernmetastasiertem Prostatakarzinom (PCa; + *n* = 23 mit WS nicht abgebildet)

Für den Anteil der Patienten, bei denen Informationen über die Ausbreitung der Tumorerkrankung vorlagen, erfolgte eine Aufschlüsselung nach den gewählten Therapiestrategien (55,1 % der Gesamtkohorte). Hierbei überwog der Anteil der Behandlungen mittels radikaler Prostatektomie (RP) und Radiatio bei den Patienten mit lokal begrenztem (17.034 bzw. 5127 von 28.325 Patienten, entsprechend 60,1 bzw. 18,1 %), lokal fortgeschrittenem (820 bzw. 486 von 1988 Patienten, entsprechend 41,3 bzw. 24,5 %) oder lymphogen metastasiertem PCa (2525 bzw. 413 von 3729 Patienten, entsprechend 67,7 bzw. 11,1 %). Bei den metastasierten Tumoren überwog der Anteil der medikamentösen Behandlungen (3985 von 5565 Patienten, 71,9 %). Eine RP wurde hier nur in Einzelfällen durchgeführt (Abb. 2a–d).

Klare Trends im Einsatz der verschiedenen Therapieverfahren zeigten sich hierbei nicht. Auch die Anteile der RP und der Radiatio an der Behandlung von Patienten mit lokal begrenztem Tumorgeschehen blieben im zeitlichen Verlauf weitgehend konstant.

## Änderungen in der operativen Behandlung

**Abb. 3:**
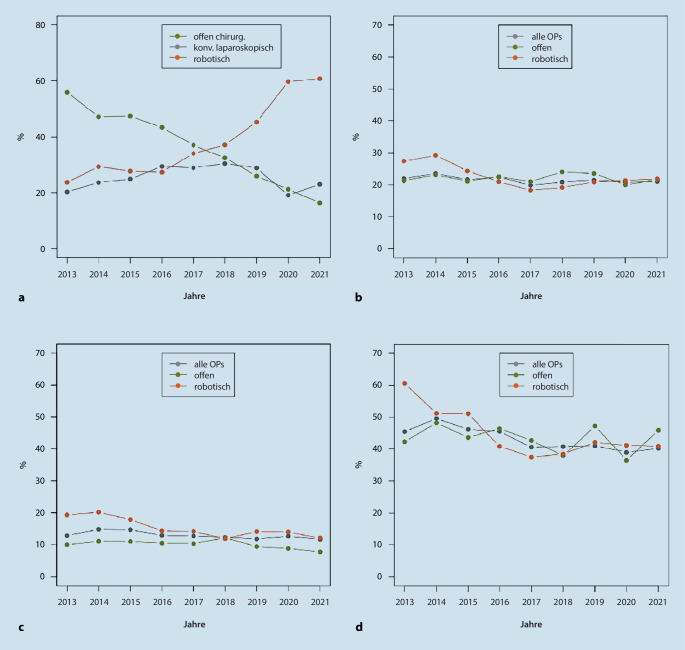
**a** Anteil der Operationsverfahren der radikalen Prostatektomie (RP) von 2013 bis 2021 (*n* = 26.745). **b** Entwicklung der R1-Raten der Operationsverfahren bei lokal begrenzten Tumoren (*n* = 16.154), **c** bei Tumoren mit pT1/2 (*n* = 12.631) und **d** bei Tumoren mit pT3/4 (*n* = 5992)

Veränderungen zeigten sich allerdings bei den operativen Zugangsverfahren zur Durchführung der RP. Hier nahm der Anteil der offenen retropubischen RP sukzessive ab (2013: 1086 bzw. 56,0 %, 2017: 1129 bzw. 37,1 %, 2021: 601 bzw. 16,3 %) wohingegen der Anteil der roboterassistierten RP (RARP) signifikant zunahm (2013: 459 bzw. 23,7 %, 2017: 1039 bzw. 34,1 %, 2021: 2240 bzw. 60,8 %). Der Anteil der konventionell laparoskopisch durchgeführten Operationen lag im Mittel bei 25,5 % und unterlag gewissen Schwankungen (19,1 % in 2020 und 30,4 % in 2018; Abb. 3a).

Die Gesamt-R1-Rate bei der RARP lag im Jahr 2013 bei 34,8 % und damit höher als im Durchschnitt aller Operationen. Im Verlauf sank sie jedoch bis zum Jahr 2017 deutlich auf 22,8 %. Hiernach stieg sie bis 2021 wieder leicht an auf 26,2 %. Im Mittel lag sie bei 26,6 %.

Bei den – entsprechend ihrem präoperativen Staging – lokal begrenzten Tumoren lag die mittlere R1-Rate bei 21,2 %. Für die RARP lag sie in den Jahren 2013 und 2014 noch deutlich über dem Gesamtdurchschnitt und der Rate bei offenen Operationen, sank jedoch nachfolgend auf Werte vergleichbar mit der offenen Operation (Abb. 3b).

Analog zum Qualitätsindikator zertifizierter Prostatakrebszentren sind in Abb. 3c die jährlichen R1-Raten bei pT1/2-Tumoren dargestellt. Die Sollvorgabe für zertifizierte Zentren lag hier bei ≤ 15 %. In BW lag die R1-Rate kumulativ bei 12,7 %. Die Sollvorgabe von 15 % wurde in keinem der untersuchten Jahre überschritten. Bei der RARP lag dieser Wert seit 2016 < 15 %. Bei pT3/4-Tumoren war der Anteil der R1-Resektionen mit kumulativ 42,8 % erwartungsgemäß deutlich höher (Abb. 3d). Abgesehen von einer in 2013 höheren R1-Rate der RARP zeigen sich hier keine relevanten Unterschiede zwischen den Operationsverfahren.

## Prognose des Prostatakarzinoms

**Abb. 4:**
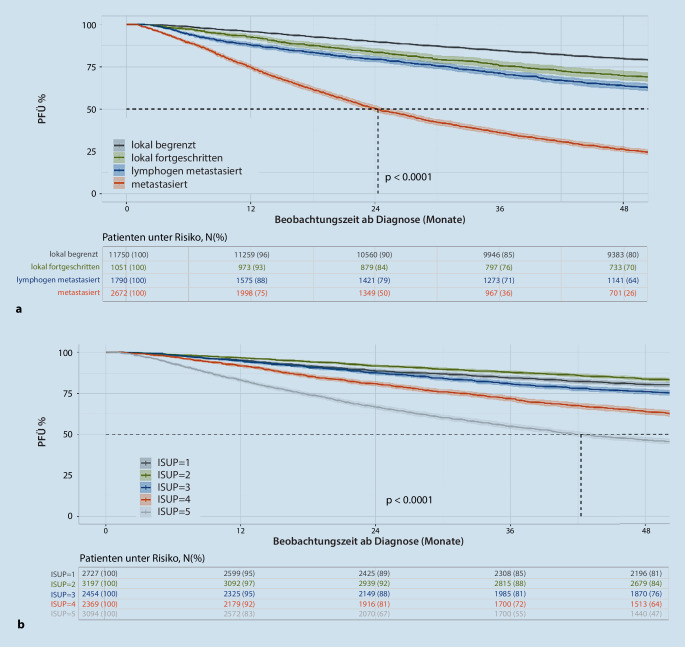
**a** Progressionsfreies Überleben (PFÜ) von Prostatakarzinom (PCa)-Patienten mit Meldedatum 2013 bis 2017 entsprechend ihrem Tumorstadium. **b** PFÜ der PCa-Patienten mit lokal begrenzten Tumoren aus dem gleichen Zeitraum entsprechend ihrem ISUP-Grading

Darüber hinaus erfolgte auch eine Analyse des progressionsfreien Überlebens (PFÜ) der Patienten, die in den Jahren 2013 bis 2017 diagnostiziert wurden (sofern ausreichende Daten zum Tumorstadium und Verlaufsmeldungen vorlagen; *n* = 17.263 von 43.530 Patienten). Die Patienten mit ED ab 2018 wurden aufgrund des kurzen Follow-up von der Auswertung ausgenommen. Hierbei zeigte sich die beste Prognose für Patienten mit lokal begrenzten Tumoren mit einem 79,8 % PFÜ nach 48 Monaten. Das 4‑Jahres-PFÜ bei Patienten mit lokal fortgeschrittenen Tumoren lag bei 69,7 %, bei lymphogen metastasierten Patienten bei 63,7 % und bei fernmetastasierten Patienten bei 26,2 % (*p* < 0,0001, Abb. 4a).

Bei der isolierten Betrachtung der Patienten mit lokal begrenzten Tumoren aus diesem Zeitraum, für die auch ISUP-Daten vorlagen (*n* = 13.841 Patienten, bei denen keine sichere Differenzierung zwischen ISUP 2 und 3 vorgenommen werden konnte, wurden ausgeschlossen), zeigte sich ein annähernd gleiches PFÜ nach 48 Monaten für ISUP 1 mit 80,5 %, für ISUP 2 mit 83,8 % und für ISUP 3 mit 76,1 %. Das PFÜ war signifikant geringer für Patienten mit ISUP 4 (63,8 %) und ISUP 5 (46,5 %; *p* < 0,0001; Abb. 4b).

## Diskussion

Die in der täglichen Behandlung von onkologischen Patienten anfallenden Daten sind sehr wertvoll im Hinblick auf die Versorgungsforschung und ein Gradmesser für die Behandlungsqualität. Die systematische Erfassung dieser Daten über die klinischen Krebsregister hat in den vergangenen 10 Jahren deutliche Fortschritte gemacht. Dennoch ist es nach wie vor eine große Herausforderung diese Daten für die Behandler optimal nutzbar zu machen. Die vorliegenden Auswertungen sollen die Behandler, neben den klinischen Aspekten, für die Relevanz einer vollständigen Meldung (Diagnose, Therapie und Verlauf) sensibilisieren, um in Zukunft noch valider Auswertungen durchführen zu können.

Insgesamt wurden im Erfassungszeitraum von 2013 bis 2021 84.347 PCa-ED an das KRBW gemeldet. Im Mittel entspricht dies 9372 Neuerkrankungen im Jahr. Der in den Jahren 2015 bis 2019 verzeichnete sukzessive Anstieg der ED ist mit der demografischen Entwicklung und der damit einhergehenden Zunahme altersassoziierter Erkrankungen erklärbar [2, 3, 8]. Die Zahlen der in den Jahren 2020 und 2021 gemeldeten PCa liegen geringfügig unter der von 2019. Dies ist möglicherweise durch einen Meldeverzug erklärbar. Andere Ursachen wie die Coronapandemie könnten allerdings auch eine Rolle spielen. Jedoch weisen internationale Daten darauf hin, dass – möglicherweise bedingt durch einen veränderten Einsatz des PSA-Screenings – in vielen entwickelten Ländern die PCa-Inzidenz stagniert oder sogar leicht sinkt [1]. In den USA hat infolge der negativen Bewertungen des PSA-Screening durch die United States Preventive Service Task Force (USPSTF) in den Jahren 2008 und 2012 die Inzidenz des PCa zunächst abgenommen, um dann von 2015 an wieder anzusteigen [9, 10].

Limitiert ist die Aussagekraft der Auswertungen per se durch den retrospektiven Charakter der Datenerhebung. Zwar stammen die erhobenen Daten im Gegensatz zu prospektiven Studien aus der tatsächlichen Behandlungsrealität, dies geht jedoch zulasten der Datenkonsistenz. Wie Eingangs beschrieben, wird dem KRBW zwar eine hohe – und extrapoliert aus den bundesweiten Daten auch plausible – Zahl an PCa-ED gemeldet. Allerdings fehlen bei einem gewissen Anteil bereits basale Daten, wie das Tumorstadium (kumulativ bei 54,3 % vollständig) und das ISUP-Grading (kumulativ bei 75,7 % vollständig). Insbesondere um Fragestellungen zu behandlungsrelevanten Endpunkten suffizient beantworten zu können, wären jedoch weitgehend vollständige Datensätze notwendig.

Um qualitativ hochwertigere Daten zu erhalten, müssen für die Meldung entsprechend dezidierte Datenfelder vorhanden sein. Der bis zum Jahre 2017 verwendete Meldedatensatz hatte nur einen geringen Umfang und war unzureichend spezifisch für das PCa. Nach klinisch-wissenschaftlichen Gesichtspunkten unvollständige Datensätze sind nicht per se ein Problem zu geringer Meldeaktivität. Die Notwendigkeit von Nachbesserungen wurde erkannt und ab Mitte 2017 ein entsprechendes Prostatamodul für die Meldung an die Krebsregister veröffentlicht. Hier sind nun zusätzliche Datenfelder vorhanden, die erweiterte klinische Analysen oder z. B. die Stratifizierung nach Risikogruppen beim lokal begrenzten PCa ermöglichen. Meldungen mit diesem erweiterten Datensatz konnten in BW ab Mitte 2018 erfolgen. Diese Ausweitung der dokumentierbaren Items geht mit dem in Tab. 1 gezeigten Anstieg der Dokumentationsraten einher. Vor dieser Zeit konnten aufgrund des Mangels an spezifischen Eingabefeldern zusätzlich Informationen nur in einem Freitextfeld angegeben werden, was die Übertragung in die Krebsregister erheblich erschwerte.

Eine zentrale Erfassung, wie es sie z. B. in Großbritannien gibt, würde dieses Problem nicht lösen. Andere große retrospektive Datenbanken wie die US-amerikanische SEER-Datenbank („surveillance, epidemiology and end results“) haben ebenfalls relevante Limitationen durch einen großen Anteil an „missing data“. Eine Analyse aus der Arbeitsgruppe von Matthew Cooperberg zeigte beispielsweise auf, dass nur bei 46 % von 257.060 Patienten mit klinisch lokalisiertem PCa (T1‑2 N0 M0) vollständige Angaben zu grundlegenden Tumorcharakteristika zur Bestimmung des prätherapeutischen Risikos vorlagen [11].

Sowohl die Optimierung der Datenerfassung als auch der Parameter und Abläufe der Meldung sind Aspekte, an denen die Krebsregister kontinuierlich arbeiten, um das Problem fehlender Daten zu reduzieren.

Dies erklärt auch, warum die hier aufgezeigten Kaplan-Meier-Analysen zum PFÜ von Patienten mit ED in den Jahren 2013 bis 2017 auf den Daten von lediglich 39,7 % der in diesem Zeitraum gemeldeten Patienten beruhen. Das Risiko für eine Verzerrung der tatsächlichen Erkrankungsrealität ist somit gegeben, insbesondere falls lückenhafte oder fehlende Meldungen nicht dem Zufall, sondern systematischen Ursachen unterliegen sollten. Ob dies so ist, lässt sich anhand der vorliegenden Daten nur mutmaßen, sodass die gezeigten Ergebnisse unter dem Vorbehalt der Annahme, es handle sich jeweils um repräsentative Stichproben, interpretiert werden müssen.

Aufgrund der vergleichsweise hohen Meldedichte erscheinen die Daten zum ISUP-Grading am verlässlichsten. Es zeigten sich hier keine wesentlichen Änderungen in der Inzidenz verschiedener ISUP-Gruppen über den Erfassungszeitraum. Insbesondere durch die oben genannte Erweiterung der Meldedetails nahm der Anteil derjenigen Patienten, bei denen lediglich „Gleason 7“ – ohne Angabe des dominanten Gleason-Patterns – übermittelt wurde, deutlich auf zuletzt < 1 % der gemeldeten Fälle ab.

Im Hinblick auf die Tumorausdehnung zeigte sich ein weitgehend konstanter Trend mit Zunahme der lokal begrenzten Tumoren von 65,5 % im Jahr 2013 auf 77,1 % im Jahr 2021 bei jeweils leichtem Rückgang lokal fortgeschrittener (6,8 % in 2013 vs. 3,3 % in 2021), lymphogen metastasierter (9,7 % in 2013 vs. 7,3 % in 2021) und fernmetastasierter Tumoren (18,1 % in 2013 vs. 12,3 % in 2021). Eine Ursache für diese Entwicklung lässt sich anhand dieser Daten nicht ablesen, sie könnte jedoch Folge einer intensiveren Vorsorge und/oder eines gestiegenen Gesundheitsbewusstseins der männlichen Bevölkerung sein [12, 13]. Daten aus den USA aus der Zeit nach der USPSTF-Empfehlung zeigen hingegen eine Verschiebung hin zu weiter fortgeschrittenem Tumorstadium, höherem Tumorgrad und Risiko [9]. Für ganz Deutschland machten in den Jahren 2017 bis 2018 Tumoren der UICC-Stadien I und II (bis maximal T2c N0 M0) ca. 65 % aus, vergleichbar zu den hier berichteten Daten [2].

Bei den Therapien der lokal begrenzten Tumoren dominiert klar die operative Behandlung. Eindeutige Trends in der proportionalen Verteilung der Behandlungsmodalitäten über die Zeit ergeben sich nicht. In 8,0 % der Patienten mit Fernmetastasen erfolgte eine operative und in 20,1 % eine strahlentherapeutische Behandlung. Ob es sich hierbei um oligometastasierte Patienten mit individuell abgestimmten multimodalen Therapiekonzepten (z. B. mit einer Radiatio des Primarius analog zur STAMPEDE H-Studie) handelt, bleibt unklar [14]. Denkbar wäre auch eine erst kurz nach Therapie diagnostizierte Metastasierung.

Eindeutig ist jedoch die zunehmende Verbreitung der RARP, welche mittlerweile als das Standardverfahren bei der kurativ intendierten Behandlung des PCa bezeichnet werden kann. Mit zuletzt einem Anteil von knapp über 60 % wird die überwiegende Zahl der Patienten mittlerweile auf diese Weise operiert. In den USA waren es im Jahre 2017 zwischen 62,0 % (Medicare) und 78,1 % (private Gesundheitsanbieter) der Operationen, in GB sogar 85,1 % [15]. Die hohen Anschaffungskosten und höheren Materialkosten pro Operation, welche lange Zeit in Deutschland nicht in der Vergütung abgebildet waren, sind als zwei der wesentlichen Gründe für die langsamere Verbreitung der Technik in Deutschland zu nennen. Besonders ausgeprägt in GB ist die Zentrenbildung für komplexe Behandlungen. In 2017 führten dort bei jahrelang sinkender Zahl nur noch 95 Krankenhäuser RP durch [15]. In Deutschland gibt es Stand Juni 2023 hingegen allein 141 zertifizierte Prostatakarzinomzentren, wobei aktuell eine Zertifizierung nicht Grundvoraussetzung für die Durchführung dieser Operation ist [16, 17].

Auch wenn es eine randomisierte Phase-III-Studie zum Vergleich der offenen retropubischen RP mit der RARP gibt, konnte diese bisher keine Unterschiede hinsichtlich des onkologischen Langzeitergebnisses aufzeigen [18]. Anhand der vorliegenden Daten ist aufgrund des noch recht kurzen Follow-up hierzu ebenfalls keine Aussage möglich. Als kurzfristiger Surrogatparameter kann die R1-Rate bei lokal begrenzten Tumoren (gemäß dem präoperativen Staging) dienen. Diese ist auch ein zentraler Qualitätsparameter bei der Beurteilung von zertifizierten Prostatakarzinomzentren. Diese lag für alle Tumoren im Mittel bei 26,6 % (2013: 27,2 % vs. 2021: 24,8 %). Unterteilt in offene und roboterassistierte Operationen lagen die R1-Raten bei 26,1 % bzw. 34,8 % in 2013 und 28,4 % bzw. 26,1 % in 2021. Bezogen auf pT1/2-Tumoren liegt die kumulative R1-Rate bei 12,7 % und somit unter der für zertifizierte Prostatakrebszentren geforderten Obergrenze von 15 %. Bei der RARP liegt dieser Wert nach anfänglich höheren Raten seit 2016 durchgehend bei < 15 %, jedoch etwas höher als bei der offenen Operation. Internationale Daten hingegen beschreiben für die R1-Rate der RARP etwas niedrigere Werte als für die offene RP und das Verfahren bietet ferner die Vorteile eines geringen Blutverlusts, einer geringeren Transfusionsrate und eines kürzeren Krankenhausaufenthalts [15, 19]. Die funktionellen Langzeitergebnisse sind vergleichbar [18, 20].

Die ausgewerteten Daten zeigen eine klare Abhängigkeit der Prognose von Stadium und Tumorgrad bei ED. Bei lokalisierten Tumoren hatten insbesondere ISUP-5-Tumoren ein signifikant kürzeres PFÜ. Daten aus einer großen Serie zur perkutanen Radiatio (*n* = 41.735, 2005–2015) zeigen ein metastasenfreies Überleben und PCa-spezifisches Überleben nach 10 Jahren von 96 % bzw. 98 % für ISUP 1, 92 % bzw. 97 % für ISUP 2, 87 % bzw. 94 % für ISUP 3, 83 % bzw. 93 % für ISUP 4 und 67 % bzw. 82 % für ISUP 5 [21]. In einer prospektiven Studie (*n* = 347, 1989–1999) zur RP beim lokalisierten PCa lag die kumulative Inzidenz für Metastasen nach 23 Jahren bei 26,6 %. 19,6 % der Patienten waren zu diesem Zeitpunkt an ihrem PCa verstorben [22]. Neuere Daten (*n* = 22.033, 2005–2015) zeigen eine 10-Jahres-Rate für biochemische Rezidive von 29 % und eine metastasenfreies Überleben von 77 % [23].

Insgesamt zeigen die Daten einen Überblick über Inzidenz und Behandlung des PCa in BW von 2013 bis 2021. Wie bereits ausgeführt, sind die gemeldeten Daten teilweise unvollständig. Die beschriebenen Daten sollten neben der klinischen Aussagekraft auch als Motivation für eine noch intensivere Meldung an die Krebsregister dienen. Zeitnahe und detaillierte Meldungen leisten einen wichtigen Beitrag zu einer umfangreichen und aussagekräftigen Datengrundlage für zukünftige Auswertungen.

## Fazit für die Praxis

- Die Zahl der Prostatakarzinomerstdiagnosen in Baden-Württemberg nimmt kontinuierlich zu.
- Der Anteil primär metastasierter Tumore nimmt ab.
- Bei der operativen Therapie lokalisierter Tumore dominiert die radikale Prostatektomie.
- Die roboterassistierte radikale Prostatektomie ist mittlerweile das häufigste Operationsverfahren.
- Nach anfänglich höheren R1-Raten ist die roboterassistierte Operationstechnik inzwischen vergleichbar mit der offenen Operation.
- Die Prognose korreliert maßgeblich mit dem Erkrankungsstadium und der ISUP-Gruppe (Internationale Gesellschaft für Urologische Pathologie) bei Diagnose.
- Zeitnahe und detaillierte Meldung sind von größter Relevanz für aussagekräftige Auswertungen.
